# Student Attitudes toward Learning Analytics in Higher Education: “*The Fitbit Version of the Learning World”*

**DOI:** 10.3389/fpsyg.2016.01959

**Published:** 2016-12-19

**Authors:** Lynne D. Roberts, Joel A. Howell, Kristen Seaman, David C. Gibson

**Affiliations:** ^1^School of Psychology and Speech Pathology, Curtin UniversityPerth, WA, Australia; ^2^Faculty of Health Sciences, Curtin UniversityPerth, WA, Australia; ^3^Curtin Institute for Computation; UNESCO Chair of Data Science in Higher Education Learning & Teaching, Curtin UniversityPerth, WA, Australia

**Keywords:** learning analytics, higher education, student attitudes, dashboards, big data

## Abstract

Increasingly, higher education institutions are exploring the potential of learning analytics to predict student retention, understand learning behaviors, and improve student learning through providing personalized feedback and support. The technical development of learning analytics has outpaced consideration of ethical issues surrounding their use. Of particular concern is the absence of the student voice in decision-making about learning analytics. We explored higher education students' knowledge, attitudes, and concerns about big data and learning analytics through four focus groups (*N* = 41). Thematic analysis of the focus group transcripts identified six key themes. The first theme, “Uninformed and Uncertain,” represents students' lack of knowledge about learning analytics prior to the focus groups. Following the provision of information, viewing of videos and discussion of learning analytics scenarios three further themes; “Help or Hindrance to Learning,” “More than a Number,” and “Impeding Independence”; represented students' perceptions of the likely impact of learning analytics on their learning. “Driving Inequality” and “Where Will it Stop?” represent ethical concerns raised by the students about the potential for inequity, bias and invasion of privacy and the need for informed consent. A key tension to emerge was how “personal” vs. “collective” purposes or principles can intersect with “uniform” vs. “autonomous” activity. The findings highlight the need the need to engage students in the decision making process about learning analytics.

## Introduction

Higher education institutions collect a wide range of electronic data (“big data”) from students (Picciano, 2012; Daniel, 2015). “Big data” may include information on student demographics, enrolments, university learning management systems, surveys, library usage, student performance, and external data sets (de Freitas et al., 2015). The collection, analysis and reporting of big data on students to predict student retention, understand learning behaviors, and improve learning through providing personalized feedback and support is referred to as learning analytics (Siemens, 2013). Big data can be used for learning analytics purposes at range of levels within the university, from university wide models predicting retention (e.g., de Freitas et al., 2015 modeled retention based on 1272 measures of behavior), through to course level data providing feedback on learning on a particular subject to individual students (Arnold and Pistilli, 2012).

The majority of universities are investigating, or are already using, learning analytics, typically with a focus on predicting student retention (Arnold and Pistilli, 2012; Corrin and de Barba, 2014; de Freitas et al., 2015). The use of learning analytics for predictive purposes is projected to expand to university- and system-wide projects (Heath and Leinonen, 2016; Roberts et al., 2016). However, at the current time the application of big data to learning analytics for the purposes of learning instruction is less common (Dede et al., 2016), typically involving small-scale projects with a focus on understanding learning and teaching practices (Siemens et al., 2013; Colvin et al., 2015). The disproportionate focus on prediction over learning highlights the gap between the use of big data and learning analytics for prediction and its application to enhancing learning (Dede et al., 2016). As argued by Dede et al. (2016), the criterion for learning analytics should be the impact on student learning, with research required into how teachers and students could use learning analytic tools to increase learning. In order to develop tools to facilitate student learning, an important first step is to understand student attitudes toward, and concerns about, learning analytics. In this article we first describe the current learning analytics landscape in relation to student involvement in learning analytics research and development. Next we outline the posited benefits and risks to students associated with learning analytics, before describing what is currently known about student attitudes toward learning analytics from the limited research that has been conducted. We then present our research on student attitudes toward learning analytics based on a series of focus groups with undergraduate and postgraduate students.

The rapid adoption and expansion of learning analytics in the higher education sector has occurred at a faster pace than the consideration of ethical issues surrounding their use (Slade and Prinsloo, 2013; Swenson, 2014). Within the Australian higher education context, the “relative silence” (Colvin et al., 2015) on ethical issues has been noted. Of particular concern is the absence of the student voice in decision-making about learning analytics. Involving students as collaborators in decisions about big data and learning analytics has been recommended as a general ethical principle (Slade and Prinsloo, 2013; Roberts et al., 2016) but is seldom realized. Neglecting student involvement in the decision making process may pose challenges to the acceptability of learning analytics systems (Beattie et al., 2014). Learning analytics systems may be seen as a risk to academic freedom where students perceive they no longer have the ability to autonomously negotiate their learning environment, instead feeling forced to use a system designed by undisclosed “others” (Beattie et al., 2014). Not valuing student input also serves to foster skewed power relationships within higher education settings and frames learning analytics as a means to achieving institutional aims rather than serving students' learning (Slade and Prinsloo, 2013; Beattie et al., 2014). Neglecting the student voice also undermines transparency, autonomy and informed consent (Slade and Prinsloo, 2013; Beattie et al., 2014; Prinsloo and Slade, 2014).

To satisfy ethical guidelines and create a respectful learning environment student involvement in decision making process is necessary (Slade and Prinsloo, 2013; Beattie et al., 2014; Prinsloo and Slade, 2014). Students should have an active voice in determining what data is collected about themselves, how it is used and stored, who will have access to the data and how student identities will be protected (Slade and Prinsloo, 2013). Despite this necessity, there are few studies that have extended beyond surface level collaborations (Liu et al., 2015), predominately focusing on examining student preferences for analytic features (Atif et al., 2015; Reimers and Neovesky, 2015; McPherson et al., 2016).

The increasingly competitive nature of higher education and pressure to quickly fulfill government demands in creating nationally and globally competitive graduates may serve as an explanation for the rapid expansion of learning analytics without student involvement (Daniel, 2015). Furthermore, decreased government funding, increased tuition costs and declining admission rates combine to pressure universities to exceed their competitors and entice students with the provision of new and “best teaching methods,” in this case learning analytics (Thornton, 2013).

To date, universities have predominately focused on the role of learning analytics in fulfilling institutional aims such as institutional performance assessment, financial planning, recruitment and admissions tracking, and student retention (Daniel, 2015; Hoel et al., 2015). Learning analytic data is used by universities to enact informed change to improve institutional efficacy and effectiveness (Drachsler and Greller, 2012; Greller and Drachsler, 2012; Daniel, 2015). Despite the institutional focus, a range of benefits of learning analytics for students have been posited (Siemens and Long, 2011; Greller and Drachsler, 2012; Pardo and Siemens, 2014). Learning analytics have the potential to provide students with insight into their own learning habits, with the self-evaluation of data considered critical in obtaining self-knowledge (Greller and Drachsler, 2012). Higher education learning analytic systems can facilitate informed decision-making by students, allowing them to alter their learning strategies accordingly (Slade and Prinsloo, 2013). Learning analytic systems are also proposed to improve the feasibility of effective early intervention strategies (Greller and Drachsler, 2012; Pardo and Siemens, 2014), with predictive analytics enabling timely and personalized interventions to support struggling students before negative outcomes such as failing occurs (Slade and Prinsloo, 2013). Interventions may include specific recommendations for improvement (Siemens and Long, 2011) facilitated by the mapping of student activity and student profiles. Analytics could form the basis for directing resources relevant to students' learning goals and current knowledge of the topic (Siemens and Long, 2011). Such an approach provides personalized learning (Drachsler and Greller, 2012).

Despite these posited benefits, there are also risks for students associated with learning analytics. Perhaps the most important of these is that the prediction of at-risk students risks profiling students and creating self-fulfilling prophecies (Greller and Drachsler, 2012; Beattie et al., 2014; Willis and Pistilli, 2014). While there has always been the potential for teachers to profile students based on observable characteristics, learning analytics provides a wider range of student characteristics for profiling. Making judgments based on a limited set of parameters creates a context for profiling, and profiling can result in limiting students' potential and damaging self-efficacy (Greller and Drachsler, 2012). For example, data showing that students from a particular suburb struggle with comprehension skills could be used to facilitate appropriate support interventions or could result in stereotyping and discrimination based on student demographics (Greller and Drachsler, 2012). Further, while the results from predictive analytics can be used to “nudge” students toward learning activities that increase the probabilities of learning success (see, for example, Martinez, 2014), there is the potential for nudges to turn into “shoves” (increasing requirements) or “smacks” (restricting activities), decreasing student autonomy over their learning (Desouza and Smith, 2016). At risk identification also positions the students as being “wrong” (Liu et al., 2015) and may create self-fulfilling prophecies where students withdraw (Willis and Pistilli, 2014). At risk-identification may foster negative student constructions and prevent the identification of teaching and institutional deficiencies (Liu et al., 2015).

Learning analytics also poses risk to student privacy and sparks debate over issues such as data ownership (Greller and Drachsler, 2012). Questions posed include what data is collected? Who has access? How will data be de-identified? And how long does the data remain accessible? (Slade and Prinsloo, 2013). Limited research has been conducted with students concerning privacy in learning analytics (Drachsler et al., 2015) but theorized risks are linked to profiling, stereotyping and poor acceptability of learning analytic systems (Greller and Drachsler, 2012).

Students are also at risk of being involved in learning analytics without their consent, or upon providing uninformed consent. In one study, none of the nine students interviewed recalled providing consent for their university to use student-generated data from the learning management system (Fisher et al., 2014). It appears students may be overwhelmed with administrative paperwork when beginning university, transparency of university data usage is poor, or potentially both. Each creates a context where students may provide uninformed consent to participation in learning analytics.

As discussed, limited research has examined student perceptions of learning analytics. Research in this area to date has focused on student attitudes toward dashboards and alert systems (Corrin and de Barba, 2014; Atif et al., 2015; Reimers and Neovesky, 2015). Learning analytics are typically displayed to students through a dashboard. A dashboard provides a consolidated view of multiple sources of data used to deliver feedback, direct students toward resources and provide performance indicators (Corrin and de Barba, 2014). It is theorized that dashboards can be used by students to self-regulate learning based on feedback (Corrin and de Barba, 2014). Feedback enables students to monitor the progress of their learning goals and if needed, adjust their strategies for achieving those (Butler and Winne, 1995). Dashboards provide students with timely, or depending on the system, real-time feedback (Pardo and Siemens, 2014) providing students with increased opportunities for feedback compared to traditional methods such as waiting for assignment feedback. Dashboards are used to create more opportunities to engage in self-regulated learning.

Research to date provides some support for the role of dashboards in promoting self-regulated learning and motivating students. In a longitudinal study Arnold and Pistilli (2012) tracked three groups of first year university students using the Course Signals (“traffic light”) dashboard via anonymous user feedback surveys and focus groups. The majority of students reported a positive experience (89%), increased motivation (74%), and a desire for the system to be expanded to other units (58%). However, student feedback also indicated a desire for more detailed feedback up-dated in real-time and communicated through other media such as emails or text (Arnold and Pistilli, 2012). Another study sought to examine the usefulness of their dashboard on student's self-reflection, awareness and sense making (Santos et al., 2013). Students reported the dashboard helped them assess how they were performing in the course and their position in the cohort, however did not aid with time management or direction toward needed resources. However, contrary to Arnold and Pistilli (2012) students' motivation did not increase (Santos et al., 2013). Two further studies have reported that dashboards improved students self-assessment, self-efficacy, and satisfaction with the course, however did not affect grades (Kosba et al., 2005; Kerly et al., 2008). Differences in dashboards features and designs may account for differences in findings. In their review of 15 dashboards, Verbert et al. (2013) noted that only four dashboards have undergone evaluations linked to learning processes, highlighting the need for further longitudinal research in this area (Verbert et al., 2013; Gašević et al., 2015). A further body of research has focused on dashboard features (e.g., Reimers and Neovesky, 2015; McPherson et al., 2016), outside the scope of this article.

Dashboard systems can be complemented by early alert systems that provide information to teaching staff and students of potential difficulties faced by the student (Atif et al., 2015). Three studies have examined student attitudes toward early alert systems. Atif et al. (2015) surveyed 85 predominately first year university students, reporting the majority (90%) wanted to be contacted immediately when their performance in a unit become unsatisfactory, an assignment was missed or their participation was low. Students preferred contact via email rather than face to face contact and wanted to be informed of where to seek help (Atif et al., 2015). Similarly, Reimers and Neovesky (2015) reported students supported the use of automated alerts in their survey of university students. Corrin and de Barba (2014) examined how students interpret and act upon early alerts/feedback delivered via dashboards. Survey and interview data indicated most students used the dashboard as a means to reflect on their performance, as a way to create new or amended study plans and as a source of motivation. Students also reported that they liked the ability to compare their performance with peers. However, at times this would obscure success goals, for example, those desiring a high distinction would be satisfied with a distinction if it was above the class average (Corrin and de Barba, 2014).

As described above, the limited research on student attitudes toward learning analytics has largely focused on student support for dashboards and early alert systems (Arnold and Pistilli, 2012). It is important to note the novelty of the field (de Freitas et al., 2015; Slade and Prinsloo, 2015), the reported recruitment difficulties and low responses rates to surveys (Corrin and de Barba, 2014; Atif et al., 2015) and the focus on first year students (Arnold and Pistilli, 2012; Corrin and de Barba, 2014; Atif et al., 2015; Sclater, 2015b). Little is known about how attitudes may vary across years of higher education, or student attitudes toward potential ethical issues associated with the use of learning analytics.

Key ethical issues related to the use of big data and learning analytics are privacy, consent, and how data is used, stored, and protected and acted upon (Alexander and Brown, 1998; Cumbley and Church, 2013; Rubel and Jones, 2016). Slade and Prinsloo (2015) hosted an online forum posting nine questions designed to elicit discussions related to these ethical issues. Fifty university student representatives engaged in the discussion. Generating the most posts was the issue of transparency: students indicated the university could make an increased effort to inform them of what data is collected, for what purpose, how it is used and who would have access to this. Students demonstrated a clear desire to be, and to remain, informed and expressed the need for governance with a strong ethics base. Students viewed their data as valuable and needing protection via mechanisms such as opt in/out options and informed choices. Students also expressed concern about learning analytics used alongside personal tutor support during a discussion about how to best support the student experience. Students were concerned tutor involvement could lead to (mis)labeling and bias that could impact negatively upon tutor-student relationships. These findings highlight the importance of involving students early in the decision making process about big data and learning analytics in order to develop “student-centric” approaches that meet students' learning needs (Kruse and Pongsajapan, 2012; Slade and Prinsloo, 2013; Gašević et al., 2015). Slade and Prinsloo (2015) acknowledged the views expressed in the forum cannot be taken as representative of all students, but the rich contextual data found highlights the need for further research in this area.

The current study builds on the limited previous research to explore students' knowledge, attitudes and concerns about big data and learning analytics. To address the previous noted limitation of research focusing on first year students, separate focus groups were conducted with first, second, and third year students, enabling an examination of similarities and differences across year groups. The results from this research can be used to inform the development and implementation of learning analytics programs in higher education, ensuring learning analytics are developed, and delivered in a manner that is acceptable to students.

## Methods

### Participants

To better understand student perceptions of learning analytics, four focus groups with current undergraduate and postgraduate students were conducted at a large metropolitan university in Australia. Across the focus groups there were 38 undergraduate students and 3 postgraduate students from Curtin University aged 18–47 (*M* = 23.63, *SD* = 6.88). The first focus group involved five female first year psychology students aged 18–24 (*M* = 21.2, *SD* = 3.03). The second focus group comprised 15 second year psychology students aged 18–47 (12 women and 3 men, *M*_*age*_ = 24.4, *SD* = 7.87). Participants in the third focus group were 14 third year psychology students aged 19–44 (10 women and 4 men, *M*_*age*_ = 24.21, *SD* = 7.74). The final focus group involved seven students, from a range of disciplines and years across the university, aged 18–30 (3 women and 4 men, *M* = 22.57, *SD* = 5.16). Participants for the first three focus groups were recruited through an undergraduate psychology research participant pool and received participation points. Participants for the final focus group were recruited via posters and flyers distributed around the university campus and snowballing. To recompense the time commitment required, focus group four participants were provided a cash payment of $25.00.

### Materials and procedure

The research was approved by the Curtin University Human Research Ethics Committee (RDHS-37-16/AR01). Data was collected through four audio-recorded focus groups conducted by the research team and transcribed verbatim. After providing written informed consent and a definition of learning analytics, participants were asked about their current knowledge of learning analytics prior to watching brief videos on learning analytics and student dashboards in higher education (Teaching with Technology, 2013; Sclater, 2015a). The videos were presented as examples of learning analytics systems. Students were also provided with information on the current state of learning analytics within their own university. Participants discussed reactions, perceived advantages, and concerns about learning analytics in response to the videos, information on dashboards, and a series of learning analytics scenarios that depicted dashboards and possible automated or teacher-generated learning analytics alerts. As participants discussed their reactions and perceptions about learning analytics, the facilitators (LR, JH, and KS) also used prompts such as, “what would that [concept] mean for you?” or “can you tell me a bit more about that [concept]?” to better understand student views without changing the potential meaning of the students discussion. Focus groups lasted approximately one and a half hours. After each focus group LR and JH discussed the key findings emerging.

Once transcribed focus group data were imported to NVivo (Castleberry, 2014) and subject to a thematic analysis, according to the procedure outlined by Braun and Clarke (2006). Following data familiarization, data was sorted into starting nodes of attitudes, preferences, misconceptions, and concerns, with further child nodes (representing codes) generated using an inductive process during coding. These codes were then grouped to develop overarching themes. The initial coding and theme development was conducted by KS. Themes were further refined through revision of transcripts and team discussions (LR, JH, and KS). During these discussions, relationships between themes were identified and a series of thematic maps depicting these relationships were created to aid the discussion and finalization of themes.

There are two indicators of the adequacy of the sample and the themes developed. First, four focus groups comprising 41 participants were conducted. Previous research has suggested that 80% of all themes can be identified in two to three focus groups (Guest et al., 2016). Second, we systematically sampled first, second, and third year students respectively from one degree for the first three focus groups to ensure we could identify possible similarities and differences across cohorts, and followed this with a focus group comprising students from varying degrees and years. No new themes emerged from this final focus group, suggesting that we were approaching saturation.

### Findings

Six key themes emerged from the analysis. The first theme, “Uninformed and Uncertain,” represents students' views on learning analytics at the commencement of the focus group. The remaining themes emerged following the provision of information, viewing of videos, and discussion of learning analytics scenarios. Three of these themes; “Help or Hindrance to Learning,” “More than a Number,” and “Impeding Independence”; relate to students' perceptions of the likely impact of learning analytics on their learning. The two remaining themes; “Driving Inequality” and “Where Will it Stop?” represent ethical concerns raised by the students. Each of the themes is expanded upon below.

#### Theme: uninformed and uncertain

The theme “Uninformed and Uncertain” reflects that most students were unaware or unsure of what big data and learning analytics were at the start of the focus groups. This was reflected in one student's comment that, “*I hadn't heard of it until today*.” Not only did some students explicitly state they were unsure of what learning analytics was, even those students who offered ideas of what it might be relied on speculative language when offering responses, for example: “*Well, like, it might show what services are needed, so if you have, like, a large population in certain areas; you could get, like, extra help in these areas*.” Learning analytics was seen as aligned with improvements in technology, “*Well, that's the way the world is going. It's become—technology is making analytics so relevant*.”

Although, students were uncertain about what learning analytics was, a few students tentatively suggested that there may be benefits for higher education students and institutions. For example, one student reported learning analytics might be useful “*in designing how to teach certain units like, to suit everyone's learning styles*,” while another student reflected that “*I think they* [Universities] *use it to improve the student experience*.” One view expressed was that learning analytics may be used to benefit the institution economically: “*Or if you can fit, sort of, extra people in, that's more people paying for the class and that sort of thing*.” Other students thought that the higher education institution could use learning analytics as a way to determine where the institution should allocate resources. One student reported:

*You can like, look at it and apply what sort of, facilities are more needed than others so instead of putting a ton of money into one area that normally we use, and you can't take away from them into an area that needs more funding*.

As students speculated about learning analytics, two concerns about the use of learning analytics emerged. Students were concerned about who would have access to their information; “*I think that the main concern would probably be privacy*”; and that learning analytics could bias their treatment in a higher education institution, for example: “[if] *a person who's marking your work gets your results—your blackboard, log in amounts, and stuff like that. And it's—and it's like, ‘Oh this person doesn’t do enough from blackboard.' …that could affect their marking*.” Even when students were not certain about what learning analytics entailed, once students began discussing how learning analytics could be used, many were quick to consider the functional impact on their educational experience.

Once students were provided with more information about learning analytics, their ideas developed and they were able to articulate a range of views and concerns about learning analytics, reflected in the remaining themes. The concerns about privacy and bias evident in this theme are explored again later in the themes “where will it stop” and “driving inequality.”

#### Theme: more than a number

The theme “more than a number” captures students' reflections on the potential for learning analytics to provide a more personalized experience. Students reported currently feeling relatively anonymous within their courses: “*I already feel like, there so many students in every course, they're already like a faceless number to some extent.”* The presentation of individualized learning analytics was viewed by some as having the potential to acknowledge a student as a person rather than a number. Students perceived that if teaching staff are able to identify students who were doing well, this may aid in the establishment of personalized relationships.

…*it kind of helps the tutor identify people that like, have the potential to do really well, they'll say, “Oh look, you're already doing really, really well on your own.” You're not being shown any favouritism but—at this point I'm like, just gonna, yeah, can send you a message, and I'm like, “Oh, yeah. I think you can really get something out of this book.”*

This was not seen as restricted to only those who are doing well. The importance of establishing a relationship is also reflected when students considered those performing poorly:

I think any measure to personalize a learning experience especially in the big university like Curtin, it can only be a good thing, I think if I was in danger of failing a unit and I had a tutor send a personalized message going, “I see you're struggling, come and see me,” that would be a good thing, I think.”

Here the student suggests a personalized message would help create a relationship between themselves and the tutor, thus encouraging them to seek assistance.

#### Theme: help or hindrance to learning?

This theme represents students' views on how learning analytics might impact on their learning. Students' positive attitudes toward learning analytics were underlined by an attitude that collecting more information could only be of benefit: “*I think, big data, I think it increases the chance of accuracy*.” Students identified that learning analytics may help teaching staff identify students who have not performed well in previous or current units, and that they could use this information to offer more support: “*Helpful to the lecturer to kind of go, ‘Okay so there’s a group of students that are doing really badly in these areas.'*” It was also suggested that emails about poor performance include information on support services: “*an automated email could be sent out to those people saying you've been identified in this zone, we're here to help you. These are the options available to help you, feel free to come and see us.”* Interestingly, the majority of students reported a preference for automated emails over emails from teaching staff, and this was seen as an equity issue: “*you shouldn't be getting like a personalized message when other students aren't.”*

Students anticipated that the unit coordinators would see not only what resources were accessed, but how frequently these resources were accessed, allowing them to continually improve the content offered in their unit. However, students also noted the potential for learning analytics to collect, display and use information that did not accurately reflect their learning activities. As one student commented, “*there's information how long you've been on Blackboard and how the books you got out. There—there's like a risk of the data not being accurate,”* which identified that a student's Blackboard login or borrowing of a book does not mean the student actually engaged with activities on Blackboard or read the book. Students' also expressed concern about their performance being predicted based on past cohorts of students, “*I think each student is different so I don't know if it's right to predict from past students.”*

Conversation focused on the personal gains each student may experience as a result of learning analytics. In particular, leaning analytics was seen to have the potential to improve motivation: “*It's kind of like, the fitbit version of the learning world that it's tracking your progress and rewarding you for, you know, for doing well, and telling you to keep it up*.” The ability for learning analytics to be used to target opportunities to students based on performance was largely supported: “*There's a feeling of being awarded.”* Similarly, some students expressed that it could be useful to identify when they need to do more work, for example:

It's a good wakeup call. If you haven't been going to classes whatever and you're like, “That's fine, that's fine.” And then you look at that and you see a correlation between, “Oh man, my grades have dropped down. I haven't been going, like and when you see it in paper, that's when you sort of like, ‘Oh, okay, yeah.”’

First year students, in particular, viewed learning analytics as providing a directed learning experience, providing feedback on how they are going, where they needed to focus their efforts and referral to appropriate resources. This arguably reflects their transitioning phase from high school to university. However, a third year psychology student also noted the advantages of directed learning through learning analytics,

For example if you're back and you're struggling a bit and you have a meeting with the lecturer and they say, “Well we can actually see what you've accessed and perhaps we can explain it. It's because you missed tutes four, five, and six and lectures one, two and three that you've struggled. We could think that the way for you to improve is to attend your lectures and tutorials perhaps” or “This piece of vital information was presented in this tutorial and you didn't go.”

Students appreciated the role of learning analytics to keep them informed. For example, when discussing the potential for alerts to be sent to students who are eligible to apply for scholarships one student stated,

I think that's a really good thing. Because there's a lot of scholarships that are available that students aren't aware of like—unless you actually go and look for it. There's a lot that don't even get claimed just because people are unaware that they are eligible for them.”

The potential for learning analytics to provide data enabling students to compare their academic performance with their peers was more contentious. Some students would value this opportunity: “…*you might not have much of a clue on how you're going, so that would, I guess, demystify that area*.” This was seen as important for students who do not attend campus frequently: “…*it can feel pretty isolated at times where you don't know what's going on or how everybody else is traveling*.” The ability to clarify where the individual sits within their cohort appeared to be valued toward the end of student's degrees:

Especially if you want to get into honors, you'd be like, “You know maybe I need to be putting more up into this unit.”

However, not all students were in favor of receiving information that compared their performance to the performance of peers. As one student stated “*I don't think that peer aspect is necessary. I think it should be more directed at your performance and really it's like an individual assessment and not so a comparison between everybody doing this*.” This view was most widely held by the first year students, who related it back to their high school experience, “*I think you get a little bit tired of ranking actually after Year 12. That was all anyone ever cared about—the ranking—just no, had enough of it.”* Concern was raised that this practice could be divisive; “*it isolates like an upper tier of students, there's kind of like that competitive fire between the students rather than a sense of community*”; and work against the current student culture that was accepting of diversity: *One of the main incentives for me coming to Curtin was it is more accepting, it has a wider demographic of students*.

Students discussed how learning analytics information displayed on dashboards or sent to students through alerts may have unintended negative consequences. Receiving information that a student is not doing well in their studies may impact negatively on their emotions, student identity and future learning: “*Probably like dejected…might give up and drop the whole course or unit”* and “*like maybe I'm not fit for the course or something.”* Even where support or additional resources are suggested the result may be negative: “*For someone who is not doing well, and to get told about things, …can be too overwhelming.”* The likelihood of a negative reaction was seen as more likely for students who were working hard in their studies:

*For someone who struggles with concepts and is putting a lot effort and yet still not making the grade. It's—it could probably quite disheartening and in turn make it a lot harder for them to have the commitment to try even harder to reach that grade*.

Students also raised the possibility that learning analytics could pressure students who are not suited to a particular degree or studying to remain with the university:

*I can see there is a potential for universities to use this just to keep students on as long as possible while accruing* [funding] *and having tax payers pay the university degree, when maybe they just might not be suited to university*.

Potential negative consequences were also suggested in circumstances where students were performing well, with suggestions that motivation, studying behavior, and performance might suffer. Some students suggested this might take the form of reduced effort: “*you might slack off a little bit.”* Other students reported they would feel pressured to perform, particularly when the information came directly from the unit coordinator (rather than an automated message) or contained suggestions for further work, and one student commented they might feel a “*Bit pressured maybe, to keep up to that standard.”* Students also noted that if multiple messages were received “*The lecturer's continuously watching you is pressuring.”*

It is not only students who are doing extremely well or poorly that might be affected by learning analytics. Some students predicted they would experience pressure from the continual display of grades and participation in dashboards, “*I think I'll be really stressed, like reflecting my attendance and participation and every single score.”* Personality was also suggested as a factor that may influence how students react to learning analytics information:

*I would imagine an anxious person receiving a bad signal on that, like, I know someone that I study with now, and she's just a stress head –…She'd flip out, she wouldn't sleep for days*.

However, it should be noted that not all students expected to have any emotional or behavioral reaction that differed from the current situation when students find out how they are doing in comparison to other students: “*I don't think I'd be—I'd feel any different to what I would feel now when people talk about their marks.”*

#### Theme: impeding independence

The theme “Impeding Independence” represented a tension students expressed that while they appreciate the additional supports that learning analytics could offer, the students valued being in charge of their own education. Several comments reflected this view, such as, “*I can handle my own education*” and “*Education needs to be—going on your own merit yourself*.” The desire to have control over one's education was fostered by the differentiation between secondary and tertiary learning expectations:

*I think that's fair in primary and secondary education but when you go to a tertiary institution you presume that because you wanna learn…you shouldn't have people say, “Oh you need to this, you need to do that,” like, you should be—we're adults, you should be held accountable for your actions*.

Students appeared to be concerned learning analytics would diminish the expectation to be self-reliant and create an environment where students are no longer treated as adults:

*We're not here to be babied, we're all like you've got to be self-motivated. There's got to be an element of initiative when you are at university. You can't expect somebody to hold your hand all the way through it*.

Students reported they were aware of what was expected and it was their own responsibility to manage the work; “*I don't feel like you need to constantly be told about it. You need to watch this lecture, you need to attend this tutorial. It's common sense, we're adults essentially”;* and seek further assistance if required: “*I know what the reading is …if I wanna do further reading, I will pursue that myself or I will ask my lecturer for what information that I could read.”* Each quote demonstrates students' reluctance to be “babied” and the desire to self-direct their learning; something they fear will be removed if learning analytics becomes a way to “micromanage” students.

Students further noted that much of the learning analytics information, such as grades and comparison to peers, was already available to them through other means, making learning analytics redundant. As one student commented when looking at an example dashboard:

*We can see that anyway with the line information that they give you on the bell chart* [when marks are released], *so you can see if you're in the top ten percent, and it's no different to what information is already out there*.

Students were wary that the over-dependence on learning analytic systems at university could then become a problem when students enter the workforce where similar systems may not exist:

…*in all likelihood if you have a professional job, you're not going to be having someone hovering over your desk telling you about, you know looking at your every keystroke seeing whether or not you're doing any good, and sort of every month pulling you aside and telling you specifically what exactly you…you know you do have to sort of gain a level of self-awareness and responsibility to sort of tell for yourself how you are doing*.

#### Theme: driving inequality

The theme “driving inequality” stemmed from students noting that learning analytics may result in only some students being advantaged. Students considered potential ethical implications learning analytics may pose. Specifically, students raised concerns about equity and bias.

##### Equity

Students highlighted an underlying tension regarding the use of learning analytics. Although, students identified that they would appreciate personalized or automated messages indicating they are performing well in comparison to their cohort or providing information on additional resources, this was seen as inequitable: “*you shouldn't be getting like a personalized message when other students aren't.”* Students were also concerned extra guidance from coordinators would unfairly impact on student grades, “*if he gets that email and that influences his overall grade, did everyone else get that email?”* Students described how they would feel annoyed; “*I would be complaining”;* or discouraged if they found out others had received an email and they had not: “*Could be a self-fulling prophecy. …—Oh, I didn't get the extra readings—oh, I think I'm dumb, I must be dumb.”*

##### Bias

The greatest concern raised by students was the potential for staff to form preconceived judgments of students and biased opinions based on learning analytics. Students were particularly concerned these biases would affect how staff interacted with them and their chances of future studies: “*If they start a class knowing that someone is likely to fail, they might not just bother putting as much effort into that because they got such a track record of having low grades”* and “*there could be preconceived judgment about my abilities to be able to complete or do something, which may inadvertently make me singled out from being available to do something.”* Concern extended to students who have performed well monopolized teaching staff's attention: “*if a teacher can see your grades they might just pay attention to the one who's getting high grades and not everyone else.”* Students clearly disliked staff being able to link their identity with their grades and online activity in fear of being treated differently or affecting future study opportunities.

#### Theme: where will it stop?

The theme “Where Will it Stop” reflects students' concerns that learning analytics may represent an invasion of privacy, and the perceived importance of obtaining informed consent from students for the use of their data.

##### Invasion of privacy

A prominent sub-theme resonating throughout the focus groups was the potential for learning analytics to compromise students' privacy: “*I kind of feel just it's a bit. It's a bit too much. Like, it's a bit—it's very personal, it's like it's—you're—yeah encroaching on personal space.”* The invasion of privacy sub theme was particularly evident when discussing the potential range of data that could be included in data-analytics in the future: “*like if I'm in my personal time, I don't really want that to be recorded.”* Students' also expressed a level of discomfort with learning analytics, “*I'll be like, a little bit, sort of weirded out, because that I know, like, everything is being watched like, calculated I guess.”* It is clear students are wary and apprehensive about how much data is collected from them and who may have access to this.

Students considered receiving alerts about specific learning activities not completed as unnecessary: “*It just seems a bit invasive.”* Students linked the reminder emails with unnecessary paternalism, “*it's just like when your parents all hover over you to do every single homework.”*

##### Informed consent

Students highlighted the need for informed consent for the use of their data for learning analytics: “*you'd have to explain to every single student exactly what Learning Analytics is, what you're doing with all of their data otherwise they can't get properly informed consent.”* They noted the difficulties in assuming informed consent from documents used for other purposes such as admission:

*I signed-up for uni[versity] four years ago, I signed a document, four years, I don't know anything on that document. So I imagine even if I'm fully informed, the day you actually signed up for—I imagine a week later you've probably completely forgotten what's in that*.

Students discussed the importance of providing opt-in or opt-out consent options for learning analytics, expressing their desire to make independent decisions concerning their involvement in learning analytics. They recognized that while some students would be keen to obtain comparative data from the whole cohort; “*the people that opt in are obviously wanting to know how they're progressing”*; others may not share this interest: “*ignorance is bliss, I—just take me out of the equation, like, I don't want to know anything about it.”*

## Discussion

The aim of this research was to explore students' knowledge, attitudes and concerns about big data and learning analytics. We found the majority of students engaged in focus groups had little, if any, knowledge of learning analytics (theme, “Uninformed and Uncertain”). The lack of knowledge extended to the types of data collected by the university and its use, supporting previous findings that students are unaware of having consented to the use of their data for learning analytic purposes (Fisher et al., 2014). This finding is not unexpected given the provision of learning analytics feedback to students is in its infancy at this university. It does however point to the absence of the student voice in the development of learning analytics, a recommended ethical principle (Slade and Prinsloo, 2013; Roberts et al., 2016) that is seldom realized and a potential threat to the acceptability of learning analytics systems (Beattie et al., 2014). The absence of student involvement is perhaps not surprising given involving students as co-creators of teaching approaches, course design, and curricula is a recommended, but infrequently implemented, practice in the higher education sector generally (Bovill et al., 2011).

When students were provided with further information and time for reflection, their attitudes toward learning analytics seem to fall into “personal” vs. “collective” purposes or principles, which intersect and cross between “uniform” vs. “autonomous” activity. The intersections give rise to some troublesome areas where conflicting purposes and audiences arise. For example, in the theme “Help or Hindrance to Learning” students acknowledge that they might want to know how they compare to others and how they are progressing (reflecting previous findings that most students are interested in receiving this information; Corrin and de Barba, 2014; Atif et al., 2015; Reimers and Neovesky, 2015), while other students might not want to know. So on the principle of personal autonomy, every student should be able to choose whether to see this information or receive messages about their relative performance. Yet, as highlighted in the theme “Driving Inequality,” out of fairness, the students also want all students to be treated equitably with messages and resources, not just a selected subgroup. So the principle of personal autonomous activity needed for independence conflicts with a collective uniform activity needed for equity.

Students supporting anonymous automated emails that are triggered and unseen by the instructors illustrates an equity goal that is in accord with personal concerns. In this case, bias cannot build up in instructors; everyone hears the same messages and gets the same access to resources as everyone else. However, as indicated above, if via autonomy, some students turn off those messages or choose not to participate, inequity may follow as some subgroups get more information and resources than others. Perhaps this form of inequity is more tolerable because it has arisen due to the choices of the students rather than to structural inequities of an impersonal uniform system.

If autonomy is supported through personal choices of the student, then some inequities are likely to be formed with only some subgroups getting certain messages and resources. This raises the question of whether there is a benchmark for the line between equity and inequity that is tolerable by the uniform system in order to not impede independence. For example, if all students have the right to not participate in seeing their information or messages and their choice leads to missing out on messages, resources and help and they become disadvantaged due to their own actions, is that a tolerable inequity?

Seeing and acting on information places all actors (e.g., instructors, unit coordinators, administrators as well as students) in this same intersecting network of personal vs. collective purposes and uniform vs. autonomous action. For example, as represented in the theme “Help of Hindrance to Learning” students see the benefit of giving instructors anonymous group information that would help them teach better to all groups (e.g., students who are struggling as well as those who are high achieving). But while some students welcome the opportunity for this to enable personalized relationships (theme “More than a Number”) others do not want the instructor to know who specifically is in those groups for fear of bias and preconceptions (theme “Driving Inequality”).

The tension resulting from the intersection of students' preferences for personal vs. collective purposes with uniform vs. autonomous activity highlights the difficulty in developing uniform policies concerning the uniform application of rules and processes that can also allow for autonomous and personalized decision-making and action by each individual student. Students held concerns about invasion of privacy (theme “Where Will it Stop”), echoing “creepy” concerns held more widely about big data (Cumbley and Church, 2013). Further, some students rejected the need for learning analytics, viewing it as a retrospective step away from independence (theme “Impeding Independence”). This echoes Beattie et al.'s (2014) concern that learning analytics can pose a risk to students autonomously navigating their learning.

## Limitations and future directions for research

The findings from our research should be interpreted within the context of its limitations. First, this research was conducted primarily with undergraduate students in the health sciences. It is possible that these students may have less knowledge and understanding of learning analytics than students in other disciplines such as information technology, and may be more concerned with issues of equity and fair representation across students. Disciplinary differences in the type and frequency of assessments may also influence how students respond to learning analytics. Research across disciplines is required to understand disciplinary differences in student attitudes toward learning analytics.

Second, students were shown videos on two learning analytics systems (JISC and Purdue), and while it was noted that these were examples it may have been difficult for students to conceptualize other learning analytic approaches. Using these two learning analytic examples may have biased student discussion toward these particular systems rather than to learning analytics in general. It would be of interest to explore if student perceptions of learning analytics differ if students are presented with other learning analytic approaches. Illuminating the similarities or differences between findings, when students have different learning analytic examples, may also provide universities with a clearer understanding of what students view as beneficial or potentially harmful.

Third, the focus of the current research has been on student attitudes to learning analytics, predicated on the relative absence of the student voice in decision-making about learning analytics. The other “voice” largely absent from learning analytics decisions in universities is that of the academics who teach. Along with students, academics are an intended “end-user” of learning analytics and further research is warranted into attitudes to learning analytics held by academics with teaching responsibilities.

## Application of findings

Whilst there are no easy options in developing policies and systems that address the intersecting and conflicting attitudes held by students, the starting points needs to be engaging students in the decision making process. We echo previous calls for student engagement in decision making to ensure the acceptability of the learning analytics systems developed (Slade and Prinsloo, 2013; Beattie et al., 2014; Prinsloo and Slade, 2014). This may take the form of representation from student guilds or related organizations that represent the wider student body. The findings from this research also highlight the need to inform students about big data and learning analytics activities that are planned or taking place within the university. Related to this is the need for each university to develop policy and procedures for obtaining student consent for the collection and use of their data. Ideally, this will occur as part of developing a university-wide code of practice/ethics for learning analytics, such as that developed by Jisc (2015).

## Summary

Our research highlights the limited knowledge students have about big data and learning analytics within higher education. While students expressed an appreciation that learning analytics could provide more personalized learning experiences, they held reservations about the functional impact of learning analytics on their education and sought the ability to make autonomous and personalized decisions about their learning. Further, they were concerned about the potential inequities resulting from learning analytics, and invasion of personal privacy. The findings highlight the need the need to engage students in the decision making process about learning analytics.

## Author contributions

LR and JH contributed to all stages of the research project and writing. KS contributed to the focus groups and writing. DG contributed to the research design, interpretation of findings and writing.

## Funding

This project was funded by Curtin University Teaching Excellence Development Fund.

### Conflict of interest statement

The authors declare that the research was conducted in the absence of any commercial or financial relationships that could be construed as a potential conflict of interest.
